# Increased Adipocyte Hypertrophy in Patients with Nascent Metabolic Syndrome

**DOI:** 10.3390/jcm12134247

**Published:** 2023-06-24

**Authors:** Ishwarlal Jialal, Beverley Adams-Huet, Sridevi Devaraj

**Affiliations:** 1 Veterans Affairs Medical Center, Mather, CA 95655, USA; 2 UCDavis School of Medicine and VA Medical Center, 10535 Hospital Way, Mather, CA 95655, USA; 3 UT Southwestern Medical Center, Dallas, TX 75390, USA; huet@digitrain.com; 4 Texas Children’s Hospital and Baylor College of Medicine, Houston, TX 77030, USA; sxdevara@texaschildrens.org

**Keywords:** fat cell size, gluteal fat, inflammation, fibrosis, angiogenesis, metabolic syndrome

## Abstract

**Background and Aims:** Metabolic Syndrome (MetS), a global problem, predisposes to an increased risk for type 2 diabetes and premature cardiovascular disease. While MetS is associated with central obesity, there is scanty data on adipocyte hypertrophy, increased fat cell size (FCS), in MetS. The aim of this study was to investigate FCS status in adipose tissue (AT) biopsy of patients with nascent MetS without the confounding of diabetes, cardiovascular disease, smoking, or lipid therapy. **Methods and Results:** Fasting blood and subcutaneous gluteal AT biopsies were obtained in MetS (*n* = 20) and controls (*n* = 19). Cardio-metabolic features, FFA levels, hsCRP, and HOMA-IR were significantly increased in patients with MetS. Waist-circumference (WC) adjusted-FCS was significantly increased in patients with MetS and increased with increasing severity of MetS. Furthermore, there were significant correlations between FCS with glucose, HDL-C, and the ratio of TG: HDL-C. There were significant correlations between FCS and FFA, as well as endotoxin and monocyte TLR4 abundance. Additionally, FCS correlated with readouts of NLRP3 Inflammasome activity. Most importantly, FCS correlated with markers of fibrosis and angiogenesis. **Conclusions:** In conclusion, in patients with nascent MetS, we demonstrate WC-adjusted increase in FCS from gluteal adipose tissue which correlated with cellular inflammation, fibrosis, and angiogenesis. While these preliminary observations were in gluteal fat, future studies are warranted to confirm these findings in visceral and other fat depots.

## 1. Introduction

Obesity is now a world-wide epidemic. During the progression of obesity, white adipose tissue expands by two major processes: an increase in the number of fat cells termed hyperplasia and an increase in fat cell size (FCS) termed hypertrophy [1,2,3]. An increase in FCS has been linked to an unfavorable cardio-metabolic profile in numerous studies [1,2,3,4,5]. However, some groups have failed to confirm this increase in FCS as a determinant of an adverse cardio-metabolic profile [6,7,8,9]. A major issue in reporting FCS is the different methods used by investigators and this could explain some of the discrepancies [1,4]. Laforest et al. has provided a useful table of the three commonly used methods of collagenase digestion, histological analysis, and osmium tetroxide fixation, detailing both advantages and disadvantages [4]. 

Metabolic Syndrome (MetS) is a cardio-metabolic cluster of major global concern due its high prevalence, and it portends an increased risk for both type2 diabetes (T2DM) and atherosclerotic cardiovascular diseases (ASCVD) [10,11,12]. Whilst increased adiposity defined by waist circumference (WC) is a criterion of MetS, there is scant data on FCS and cardio-metabolic features in MetS [13,14].

Accordingly, in the present report, we investigated FCS status in gluteal adipose tissue biopsy specimens in patients with nascent MetS without the confounding of T2DM, ASCVD, macro-inflammation, smoking, or lipid drug therapy. We examined correlations of FCS with insulin resistance, adipokines, FFA levels, plasma glucose, triglyceride levels, high density lipoprotein -cholesterol, as well as biomarkers of inflammation, fibrosis, and angiogenesis.

## 2. Patients and Methods

We have previously published on this cohort of subjects with MetS, focusing on adipokine dysregulation, inflammation, and adipose tissue biology [15,16,17,18,19]. MetS participants (*n* = 20) and controls (*n* = 19), aged 21–69 years, were recruited from Sacramento County, CA using the criteria of the Adult Treatment Panel III (ATP III) guidelines as described previously [10,11]. MetS volunteers had to have at least 3 of the 5 cardio-metabolic features used as criteria [10,15]. Exclusion criteria for control subjects included current use of any blood pressure medications, elevated triglyceride (TG) levels (>200 mg/dL), and having 3 or more of the ATP III criteria. Other important exclusion criteria for all subjects which were determined by a screening questionnaire, and clinical examination and baseline chemistries included diabetes defined by fasting blood glucose level >125 mg/dL and HbA1c > 6.4%, clinical ASCVD, acute or chronic inflammatory disorders, and a history of smoking. Major medication exclusion criteria for subjects with MetS included anti-coagulants, steroids, anti-inflammatory drugs, statins, and other lipid lowering agents and angiotensin 2 receptor blockers. Additionally, all participants in the study had a high-sensitive C-reactive protein (hsCRP) level < 10.0 mg/L and a normal white cell count. The study was approved by the institutional review board at the University of California, Davis, and informed consent was obtained from all participants. 

Blood samples were taken from patients after histories and physical examinations after at least 12 h of fasting. All routine tests were performed using standard laboratory assays, while most of the inflammatory biomarkers were assayed by ELISA, as detailed in previously published reports [16,17]. Homeostasis model assessment of insulin resistance index (HOMA-IR) was calculated from glucose and insulin, as reported previously [17]. 

Subcutaneous adipose tissue (SAT) biopsy in the gluteal area was performed on control and MetS patients, as described previously [16]. Briefly, the gluteal region was sterilized, and 4 mL 2% lidocaine was injected to desensitize the area. A 14-gauge needle connected to a 20-mL syringe was then inserted at the site of lidocaine injection. Approximately 4–6 mL fat and fluid were aspirated, washed, and then placed in medium. Between three to four fragments of SAT were fixed in 10% buffered formalin after cleaning and processed for immunohistochemistry. Briefly, SAT sections were deparaffinized, hydrated, and incubated for antigen retrieval in citrate buffer pH 6.0 (Novus Biologicals, Centennial, Colorado, USA), blocked with a 2.5% goat blocking serum, and stained with H&E. Slides were viewed using 20× magnification, and images were obtained [19]. Using histological tissue, manual measurements of the diameters of 50 adipocytes per sample were performed using NIH ImageProJ software and the average used for analyses according to the recommendation of Laforest et al. [4]. Fibrosis (collagen, Sirius staining) and Angiogenesis (anti-CD31 and anti-VEGF staining in SAT) were quantified as described previously [19]. Sections were exposed to hydrogen peroxide for 5 min and then incubated with biotinylated anti-rat IgG (BD Biosciences Pharmingen, San Diego, CA, USA) or anti-rabbit IgG (BD Biosciences Pharmingen, San Diego, CA, USA) or anti-goat IgG, respectively (Vector Laboratories Inc., Burlingame, CA, USA). A Vector stain ABC kit (Vector Laboratories Inc., Burlingame, CA, USA) was applied to the tissue followed by DAB solution (DAKO). The slides were counterstained with haematoxylin. Sirius red staining was performed for fibrosis. 

Statistics-SAS version 9.4 (SAS Institute, Cary, NC, USA) was used for statistical analysis, and significance was defined as a two-sided *p*-value < 0.05. Results are expressed as median and interquartile ranges. The Wilcoxon Rank Sum test was used to compare age and metabolic characteristics between controls and MetS subjects. Adjustments for age and waist circumference were made with analysis of covariance models, applying log transformations for skewness as warranted. Trend analysis of FCS (µm) with increasing number of characteristics of MetS in subjects was evaluated using the Jonckheere-Terpstra (J-T) test. After combining the control and MetS groups, Spearman rank correlation coefficients were determined to assess the association between FCS and relevant variables. 

## 3. Results

As depicted in Table 1, the groups were matched for both gender and age. Cardio-metabolic features, used to define the MetS, were significantly abnormal in patients with MetS compared to controls, except for blood pressure.

In addition, plasma free fatty acid (FFA) levels, hsCRP, and HOMA-IR were significantly increased in patients with MetS. 

**Figure 1:**
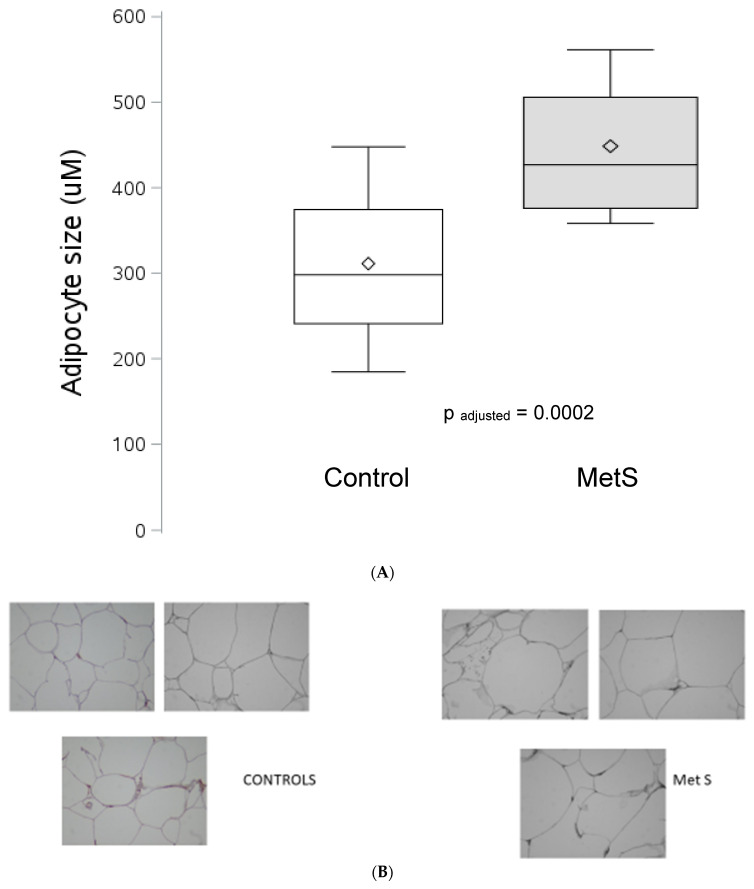
(**A**) Depiction of WC adjusted FCS in MetS and Controls. (**B**) Representative Images of AT used to quantify FCS. The blue line represents the scale bar of 50 µm.

It has been recommended that FCS be adjusted for adiposity [1,3]. In Figure 1 is portrayed the waist circumference (WC)-adjusted FCS. FCS was significantly increased in patients with MetS, *p* = 0.0002. Additionally, BMI-adjusted FCS was significantly increased in MetS. Figure 1B shows representative images of AT used to quantify FCS in controls and patients with MetS. Furthermore, FCS increased with increasing severity of MetS, defined by an increasing number of cardio-metabolic features using the J-T test, *p* = 0.0002.

Figure 1A shows the increase in FCS in MetS. The boundary of the box closest to zero indicates the 25th percentile, a line within the box marks the median, the diamond marks the mean, and the boundary of the box farthest from zero indicates the 75th percentile. Whiskers (error bars) above and below the box indicate the 90th and 10th percentiles. FCS is expressed in µM.

Correlations were undertaken between FCS and relevant variables, as depicted in Table 2. There were significant correlations between FCS and both glucose and HDL-cholesterol (negative correlation). The correlations with both WC (Table 2) and BMI (r = 0.13, *p* = 41) were not significant. This was possibly due to the small sample size, and there was a trend to significance with TG levels (*p* = 0.06). There were no significant correlations between FCS and both HOMA-IR and hsCRP. However, FCS correlated with the ratio of TG: HDL-C. Additionally, the correlations with both plasma leptin and adiponectin (*p* = 0.07) were not significant.

In a previous report, we documented significant increases in FFA, endotoxins, and monocyte Toll-like receptor4 (TLR4) [17]. There were significant correlations between FCS and the inflammatory axis of plasma FFA, endotoxin, and monocyte TLR4 abundance. In addition, FCS correlated with two important readouts of NOD-like receptor family pyrin domain containing 3 (NLRP3) Inflammasome activity (immunostaining for Caspase1 and Interleukin-1). An increase in SAT caspase 1 and interleukin-1 quantified by immunochemistry has been reported previously in this cohort [20]. 

Previously, we have reported an increase in both SAT fibrosis and angiogenesis in this cohort [19]. Furthermore, both collagen immunostaining and Fibrosis Score (Sirius Red staining) correlated significantly with FCS. Additionally, FCS correlated with both markers of angiogenesis, vascular endothelial growth factor (VEGF) and CD31. 

## 4. Discussion

We make the novel observation that, in gluteal AT, FCS, even following being adjusted for WC, is increased in carefully curated patients with nascent MetS without the confounding of T2DM, ASCVD, smoking, macro-inflammation and lipid therapy. Furthermore, FCS increased with increasing severity of MetS, defined by the number of cardio-metabolic factors.

When we examined relevant correlations with FCS, consistent with most published papers, we show significant correlations with both glucose and HDL-C levels. However, there was no significant correlation with WC, and we suggest that this could be due to the small sample size. Whilst there was a non-significant trend with TG levels, there were no significant correlations with hsCRP and HOMA-IR. Once again, these findings could be a function of the small sample size. Interestingly, we showed a significant correlation of FCS with an established surrogate of insulin resistance, the ratio of TG: HDL-C [21]. We did not observe correlations with either leptin or adiponectin with FCS in gluteal AT. Laforest et al., in 60 women, failed to show significant correlations, using the histological technique, between subcutaneous AT FCS and fasting glucose, HDL-C, adiponectin levels, and cytokines [4]. 

It needs to be pointed out that there is a serious paucity of studies on FCS in MetS, per se, especially using gluteal AT. In other subcutaneous depots, these associations have been reported with obesity [1,2,3,4]. Tittelbach et al., in a comparison of Caucasian and African American (AA) postmenopausal obese women, showed that only in AA women and not Caucasian women, gluteal FCS correlated with TG, HDL-C, and insulin resistance [13]. The majority of women in our study were Caucasian (61% Caucasian and 24% AA). This small sample size did not allow for inter-racial comparisons. In the other study, Langkilde et al. showed that, in human immunodeficiency virus-infected male patients on combined anti-retroviral therapy, FCS is increased in MetS [14]. They did not study inflammation or undertake any correlations. 

The most novel and interesting aspects of this communication is the relationship between AT fibrosis, angiogenesis, TLR4 abundance, and NLRP3-Inflammasome activity. 

It is generally considered that the increase in FCS is a function of impaired adipogenesis and cell senescence [1]. In this report, we show significant correlations with both biomarkers of increased angiogenesis, VEGF and CD31 [19], suggesting that this increase in vasculature serves as a supply of essential nutrition, such as oxygen, etc., for these hypertrophied cells. Since we did not use a functional assay for angiogenesis, we cannot conclude with certainty that these markers connote increased functional angiogenesis, and they could also reflect dysregulated angiogenesis. Villaret et al. have cogently argued for increase in premature senescence of endothelial cell in AT from their studies [22]. However, Lemoine et al. demonstrated a correlation between FCS expressed as area and the angiogenesis markers of capillary density and VEGF receptor 2 in the subcutaneous and omental AT of obese women [23]. A weakness of our report is the failure to study anti-angiogenic factors and hypoxia-inducible factor-1α, which could have been very instructive, as reviewed recently [24]. 

However, a major dictate of further enlargement of these cells is containment by the increased fibrosis due to increase in extracellular matrix components, such as collagens, etc. [25]. We show significant correlations with both increased collagen immunostaining and fibrosis score, as evidenced by Sirius red staining of AT [19]. In accord with our previous reports, we failed to demonstrate a significant correlation between FCS and macrophage density, as marked by CD68 staining (r = 0.01, *p* = 0.96). However, we showed a significant correlation with SAT mast cell abundance (r = 0.46, *p* = 0.02), which correlated significantly with both fibrosis score and angiogenesis, as documented previously [26].

The most interesting and novel observation is the strong correlations of FCS with both increased circulating FFA levels and endotoxin levels. We have previously reported elevated levels of FFA and endotoxin in these patients with MetS [17]. The increase in FFA emanated from increased lipolysis in these hypertrophied cells, which are saturated with lipid and cannot store anymore FFA. The correlation with endotoxin suggests that increased FCS could instigate gut microbiota dysbiosis by inducing inflammation. This results in an increased gut permeability and, thus, an increase in circulating endotoxin levels. Endotoxin is the classical ligand for TLR4, and FFAs appear to also act as endogenous activators, resulting in an increase in TLR-4 activity [27,28,29]. Indeed, both correlated with the increase in monocyte TLR4 activity reported previously [17]. Previously, we also reported an increase in TLR2 and 4 abundance in SAT of these patients using pooled samples for the assays [30]. However, since we pooled samples, we could not undertake correlations with FCS [30]. 

Another novel observation is the relationship with the increased AT NLRP3 Inflammasome activity that we have reported previously [20]. We show significant correlations with both caspase 1 and immunostaining for Interleukin-1. Whilst TLR4 is a cell surface receptor, its activation stimulated the formation of the NLRP-3 inflammasome complex within the cell further transducing the pro-inflammatory signals. These findings collectively underscore the relationship between FCS and cellular inflammation. 

The strengths of the present report include studying patients with nascent MetS without the confounding of T2DM, ASCVD, smoking, macro-inflammation, or lipid therapy. Another strength is the large repertoire of biomarkers/biomediators of inflammation, fibrosis, angiogenesis, and adipokines investigated. A major limitation is the small sample size. Additionally, we did not use a functional assay of angiogenesis to confirm true angiogenesis. Another weakness of our report is the failure to study hypoxia-inducible factor-1α, which could have been very instructive with respect to angiogenesis.

## 5. Conclusions

In conclusion, we show, in patients with nascent MetS, without the confounding of T2DM, ASCVD, smoking, and lipid therapy, an increase in WC-adjusted FCS in gluteal AT. The increase in FCS correlated with cellular inflammation, fibrosis, and angiogenesis. We hypothesize, based on our data, that increased FCS via inducing circulating and cellular inflammation and SAT fibrosis contributes, in part, to the genesis of MetS.

These novel findings should be confirmed in visceral and other fat depots.

## Figures and Tables

**Table 1:** Salient baseline characteristics of patients with MetS and controls.

	Controls, *n* = 19	MetS, *n* = 20	*p*-Value ^a^
Sex, Female/Male	17/2	16/4	0.66
Age, yr	50 (40–60)	55 (48–61)	0.27
BMI, kg/m^2^	28.8 (26.4–34.5)	35.4 (32.3–39.3)	0.006
Waist circumference, cm	87.6 (81.3–109.2)	106.0 (97.2–122.6)	0.0003
Weight, kg	82.7 (65.0–97.5)	98.4 (81.7–120.0)	0.01
BP-systolic, mmHg	120 (110–132)	128 (122–135)	0.14
BP-diastolic, mmHg	75 (69–82)	78 (75–85)	0.35
Glucose, mg/dL	88 (85–93)	100 (93–113)	0.0002
Total cholesterol, mg/dL	193 (162–205)	190 (167–205)	0.76
HDL-cholesterol, mg/dL	57 (47–68)	42 (37–48)	0.0006
Triglycerides, mg/dL	75 (49–109)	112 (94–160)	0.001
HOMA-IR	1.3 (1.1–2.9)	4.0 (2.4–5.8)	0.0003
hsCRP, mg/L	1.7 (0.4–4.0)	4.5 (2.2–6.0)	0.008
FFA, mmol/L	0.34 (0.18–0.44)	0.83 (0.73–0.88)	<0.0001

^a^ Wilcoxon Rank Sum test comparing Controls versus MetS for continuous variables. Results are presented—median (25th percentile, 75th percentile). BMI, body mass index; BP, blood pressure; FFA, free fatty acids; HDL, high-density lipoprotein; HOMA-IR, homeostatic model assessment of insulin resistance; hsCRP, high-sensitivity C-reactive protein; MetS, metabolic syndrome.

**Table 2:** Spearman Rank Correlation between absolute fat cell size (FCS) and relevant variables. RAU—relative arbitrary units. MFI—mean fluorescence intensity. SAT—subcutaneous adipose tissue. VEGF—vascular endothelial growth factor.

	Rho Coefficient	*p*-Values
Waist Circumference, cm	0.20	0.22
**Plasma Glucose, mg/dL**	**0.48**	**0.002**
**Plasma HDL-C, mg/dL**	**−0.48**	**0.002**
Plasma TG, mg/dL	0.31	0.06
Plasma hsCRP, mg/L	0.12	0.47
HOMA-IR	0.13	0.48
**Plasma TG:HDL-C Ratio**	**0.40**	**0.01**
Plasma Leptin, ng/mL	0.12	0.48
Plasma Adiponectin, ug/mL	0.31	0.07
**Monocyte TLR-4 (MFI/10^5^ cells)**	**0.47**	**0.01**
**PlasmaEndotoxin (EndotoxinU/mL)**	**0.68**	**0.002**
**Plasma FFA, mmol/L**	**0.63**	**0.006**
**SAT-CD 31 (RAU)**	**0.48**	**0.01**
**SAT-VEGF (RAU)**	**0.41**	**0.03**
**SAT-Collagen (RAU)**	**0.41**	**0.03**
**SAT-SIRIUS RED stain (RAU)**	**0.49**	**0.008**
**SAT-Caspase 1 (RAU)**	**0.40**	**0.03**
**SAT-Interleukin-1 (RAU)**	**0.39**	**0.03**
SAT-CD68 (RAU)	0.01	0.96

## Data Availability

The data are available from the senior author for review upon reasonable request.
